# Stomach Virtual Non-Enhanced CT with Second-Generation, Dual-Energy CT: A Preliminary Study

**DOI:** 10.1371/journal.pone.0112295

**Published:** 2014-11-13

**Authors:** Lei Shi, Fuhua Yan, Zilai Pan, Bo Liu, Huanhuan Liu, Baisong Wang, Huan Zhang, Yingyan Yu

**Affiliations:** 1 Department of Radiology, Ruijin Hospital affiliated to Shanghai Jiaotong University School of Medicine, Shanghai, China; 2 Siemens Health care, Shanghai, China; 3 Department of Biomedical statistics, Shanghai Jiaotong University School of Medicine, Shanghai, China; 4 Shanghai Institute of Digestive Surgery, Shanghai Jiaotong University School of Medicine, Shanghai, China; Wayne State University, United States of America

## Abstract

**Objectives:**

To compare the true non-enhanced (TNE) and virtual non-enhanced (VNE) data sets in patients who underwent gastric preoperative dual-energy CT (DECT) and to evaluate potential radiation dose reduction by omitting a TNE scan.

**Methods:**

A total of 74 patients underwent gastric DECT. The mean CT values, length, image quality and effective radiation doses for VNE and TNE images were compared.

**Results:**

There was no statistical difference in maximal thickness of gastric tumors and maximal diameter of enlarged lymph nodes among the TNE and VNE images (P>0.05). The mean CT value differences between TNE and VNE were statistically significant for all tissue types, except for aorta attenuation measurements (P<0.05), but the absolute differences were under 10 HU. Lower noise was found for VNE images than TNE images (P<0.01). Image quality of VNE was diagnostic but lower than that of TNE (P<0.01). The dose reduction achieved by omitting the TNE acquisition was 21.40±4.44%.

**Conclusion:**

VNE scan may potentially replace TNE as part of a multi-phase gastric preoperative staging imaging protocol with consequent saving in radiation dose.

## Introduction

Multi-detector computed tomography (MDCT) is one of the most widely used diagnostic tools for pre-operative staging of patients with gastric cancer. Standard MDCT workflow included true non-enhanced (TNE), arterial phase, and portal venous phase. The clinical practice for detection and staging of gastric masses using CT required a baseline TNE scan immediately followed by a contrast enhanced acquisition, as it depended on the diffuse enhancement and thickening of the lesion [1], [2]. However these three standard phases of scan normally derive a large amount of radiation dose which could be harmful to patients either in preoperative staging or in follow up.

Dual-energy Computed Tomography (DECT) is a promising imaging technique that provides better tissue characterization compared with single-energy computed tomography [3]–[5]. Based on two synchronous CT acquisitions at the same time, this technology allows the differentiation and identification of materials with different X-ray absorptions on low and high tube voltage [4]. This technique can differentiate attenuation of materials with large atomic numbers such as iodine-based contrast agents. On the basis of reconstruction of high- and low-kV data sets from the raw data, iodine could be extracted from a contrast-enhanced, dual-energy CT scan and virtual non-enhanced (VNE) data sets can be generated utilizing the three-material decomposition DE post-processing algorithm, which was based on the assumption of target tissue consisting of three different base materials. In the abdomen CT scan, assumption was made that contrast enhanced abdominal tissue had three base materials: soft tissue, fat, and iodine [4], [6]. The generation of VNE and iodine map distribution not only emphasized the local blood supply for lesion identification, but also allowed to avoid a TNE, which could save dose for the patient, who might benefit during both preoperative staging and oncological follow-up.

Previous studies have proposed that VNE may potentially replace TNE scans; however, most of the studies used the first generation of dual-energy scanners [7]–[12]. These first generation dual-source scanners have the limitation of a narrow field of view. Furthermore, due to an energy spectra overlap between 100 and 140 kV, these scanners had to be operated at 80 and 140 kV, resulting in the inability to be used in the abdomen due to a low penetration depth and beam hardening artifacts [13]. The recently introduced second generation dual-source scanners had overcome above problems implementing a bigger field of view (33 cm), which allowed more patients to take dual energy CT scan, and a tin filter, which reduces the X-ray spectra overlap [3], [14]–[16]. However, there were still few studies having used the second generation dual-source CT scanners that had assessed VNE has been published [11]–[12], and to the best of our knowledge, currently no previous study has been made to compare the quality of VNE and TNE images on stomach cancer.

The aim of this work was to qualitatively and quantitatively compare the image quality and noise of TNE and VNE data sets in the same patients who underwent DECT gastric preoperative examination and to evaluate potential radiation dose reduction by omitting a TNE CT scan during dual energy CT technique.

## Materials and Methods

### Patients

The study was approved by our Hospital Ethics Committee (Ruijin Hospital Affiliated to Shanghai Jiaotong University School of Medicine, involved in human research ethics committee), No 2009–34. Informed consent was obtained from each patient before imaging. Participants provided their written informed consent in this study. From April to September 2011, 74 patients (55 men, mean age of 63 years ±11 [standard deviation], range of 37–85 years; 19 women, mean age of 61 years ±11, range of 27–85 years) underwent DECT for preoperative staging of gastric cancer. All patients were histopathologically confirmed with gastric carcinoma by endoscopic gastric biopsies.

### CT scan protocol

All scans were performed using a second-generation, dual-source multi-detector CT scanner (Siemens Somatom Definition Flash, Siemens Medical Solutions, Forchheim, Germany). Each patient who had overnight fasted drank 1000–1500 ml of tap water shortly before CT to enable gastric distention. All patients underwent total 3 phase of CT scan. First, a non-enhanced scan covering the whole stomach was performed with the following setting: 120 kV, reference 200 mAs, 128×0.6 mm collimation and a pitch of 0.6. Subsequently, one hundred milliliters of a nonionic iodinated contrast agent (370 Ultravist; Schering, Berlin, Germany) was administered via the antecubital vein at 3 mL/sec using 20-gauge needle through an automatic injector. Dual energy contrast-enhanced scans were performed at arterial phase (included the whole stomach) and portal venous phase (included the whole abdomen and pelvis, from the diaphragmatic domes to the anal verge) using dual energy mode, which were acquired at 40 s and 70 s after the administration of contrast agents, respectively. The DE scans were acquired with the tube voltages at 100 and 140 kVp with tin filter, using reference mAs values of 230 and 178, respectively. The collimation was 32×0.6 mm and the pitch was 0.6. All acquisitions were obtained with real time tube current modulation (Care DOSE 4D, Siemens Medical Solutions) software.

### CT Image Post-processing

**Figure 1:**
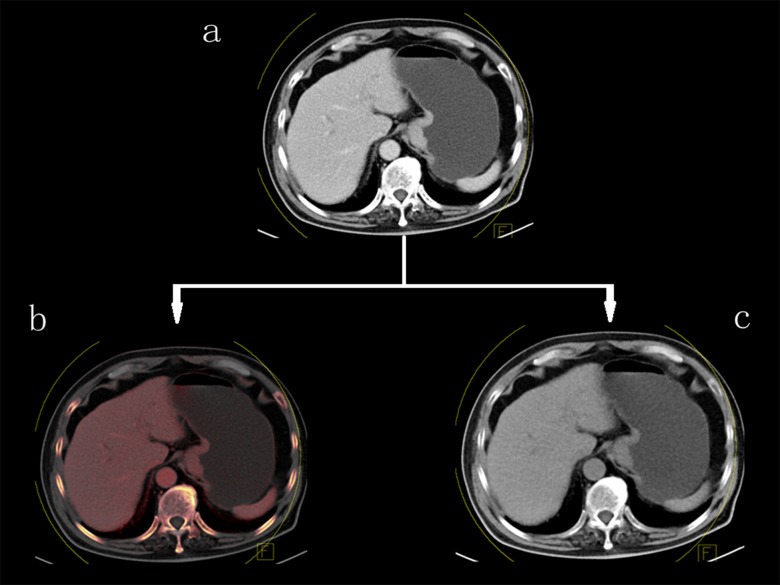
Dual-energy data post-processing. **a** Dual-energy portal venous phase image, **b** fused image of virtual 120-kV data sets with the color map of iodine distribution, **c** virtual non-enhanced image after iodine subtraction.

**Figure 2:**
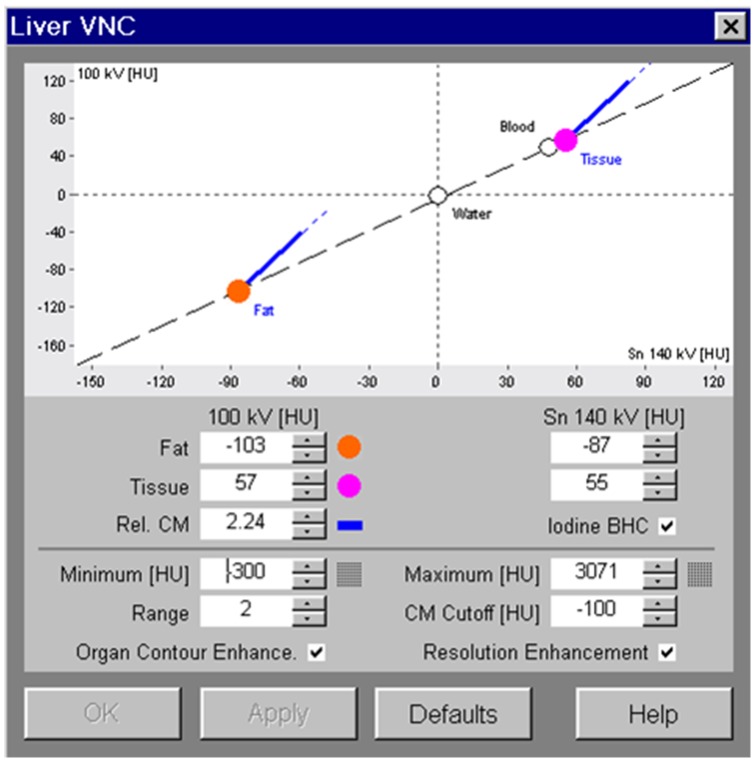
Standard values applied for analysis of soft-tissue and fat-tissue attenuation.

The DE raw data were reconstructed using a soft convolution kernel (D30f), and three different series of images were generated: 100 kV images, Sn140 kV images, and mixed images with ratio of 0.5 with a slice thickness and an interval of 1.5 mm. Images were then transferred to a DE post-processing workstation (syngo MMWP, version 2008A; Siemens Medical Solutions). DE images were processed by the “Liver VNC” application to generate iodine distribution map shown as color overlay and VNE images (Fig. 1). Axial TNE and VNE images were reconstructed by using a section thickness and an interval of 5 mm. To generate VNE images, standard soft-tissue and fat attenuation values used by the system were applied (Fig. 2).

### Image Analysis

For each patient, CT values were recorded in four anatomic regions on both TNE and VNE images: the gastric wall, liver, retroperitoneal fat and abdominal aorta by placing a circular region of interest (ROI) at each anatomical site for TNE and VNE images derived from the arterial (VNE_A_) and portal venous phases (VNE_P_). Three measurements were recorded on the same anatomic site (except aorta) at the same slice to get an average value. At all anatomical sites, a constant size of the ROI of approximately 1.5 cm^2^ was maintained except gastric wall. The ROI including full-thickness of the gastric wall was put at the antrum to get the CT value. If the lesion was located in the antrum of the stomach, the cardia was used. In patients in whom enhanced DECT detected gastric tumors or enlarged lymph nodes were visible in the non-enhanced images, their CT attenuation values and maximal thickness or diameter were measured. Standard deviations of the retroperitoneal fat were also measured to determine the image noise using a region of interest with 1 cm^2^ in area.

Two experienced abdominal radiologists, who were blinded to TNE, VNE_A_ and VNE_P_ acquisition, accessed the image quality of three sets of non-enhanced images in consensus. First, a five-point grade scale scoring system was used to score the images quality of gastric TNE and VNE images as reported previously [7]: 1 =  not assessable, not assessable due to severe artifacts or bad image quality; 2 =  poor, poor image quality due to major artifacts that hamper a complete liver parenchyma evaluation; 3 =  sufficient, image of sufficient quality that permit a good confidence in image evaluation; 4 =  good, image of good quality with minor artifacts; score 5 =  excellent, image of excellent quality without artifacts. VNE images with scores of 3 or above were regarded as acceptable for diagnosis purpose; ones with scores of 4 or more were regarded as having the potential to replace TNE images. Second, coverage of relevant anatomy by the generated VNE images was noted as a percentage range of excluded anatomy (1 =  no excluded anatomy, 2 = 1–25% excluded, 3 = 26–50%, 4 = 51–75%, 5 = >75%), in order to evaluate the influence of BMI on VNE images.

### Radiation Dose Estimation

For each of the three phases, the dose-length product (DLP, mGy cm) were recorded. Effective radiation doses (in millisieverts) were calculated for each phase by using the method proposed by the European Working Group for Guidelines on Quality Criteria in CT, applying the following relationship: E =  DLP×k conversion coefficient, where the abdominal k conversion coefficient is 0.015 mSv/mGy cm [17]. The effective dose of a triple-phase protocol (TNE CT, the arterial and portal venous enhanced CT) was compared with that of a dual-phase protocol (DE arterial and portal venous phases) to calculate the percentage of dose reduction.

### Statistical Analysis

Statistical analysis was performed using the software SPSS version 14.0 (SPSS Inc., Chicago, IL, USA). Quantitative variables were expressed as mean ± SD. Student two-tailed t test for paired samples was performed to compare the difference of CT value, image noise, radiation dose to the patients, image quality, maximal thickness of gastric tumors and maximal diameter of enlarged lymph nodes between TNE and the two sets of VNE images, respectively, and the difference between VNE_A_ and VNE_P_ images. A value of p≤0.05 indicated a statistically significant difference.

## Results

### Length, CT value and noise

Sixty-three gastric tumors in 63 patients and 112 enlarged lymph nodes in 20 patients were detected on enhanced DECT images. There was no statistical difference in maximal thickness of gastric tumors and maximal diameter of enlarged lymph nodes among the TNE and VNE images, and no difference among the VNE_A_ and VNE_P_ images(P>0.05), but lower noise were found for VNE images than TNE images, and VNE_P_ images had the lowest noise (P<0.01). All recorded image noise values were summarized in table 1.

**Table 1:** Mean Length, CT value and noise of ROI measured in three series of non-enhanced images.

	TNE	VNEA	VNEP	TNE vs VNE_A_	TNE vs VNE_P_	VNE_A_ vs VNE_P_
CT value (HU)						
tumor	36.3±6.6	34.8±7.0	34.2±6.8	0.02	0.01	0.4
LN	35.4±5.1	29.3±9.0	27.9±9.0	<0.01	<0.01	0.24
stomach	32.2±5.6	29.9±6.4	29.9±7.2	<0.01	<0.01	0.97
liver	55.1±5.7	59.9±7.0	59.7±8.0	<0.01	<0.01	0.66
aorta	42.6±6.1	42.9±8.4	40.2±6.0	0.8	<0.01	<0.01
fat	−100.0±10.2	−92.6±9.2	−92.6±9.0	<0.01	<0.01	0.99
Length (cm)						
tumor	1.67±0.75	1.66±0.73	1.64±0.72	0.78	0.28	0.46
LN	1.97±0.79	1.95±0.76	1.95±0.79	0.61	0.49	0.91
noise (SD)	9.1±2.1	4.7±1.0	4.4±1.1	<0.01	<0.01	0.05

TNE true non-enhanced CT, VNE_A_ VNE data acquired at the arterial phase, VNE_P_ VNE data acquired at the portal venous phase, LN lymph node, stomach normal stomach wall.

Although the mean CT value differences between TNE and VNE per patient were statistically significant for all tissue types, except for aorta attenuation measurements, the absolute differences were still under 10 HU. There was no statistical difference in CT values of all tissue types among the two sets of VNE CT images, except for aorta attenuation measurements (P>0.05). Mean CT values and P-values are given in Table 1.

**Figure 3:**
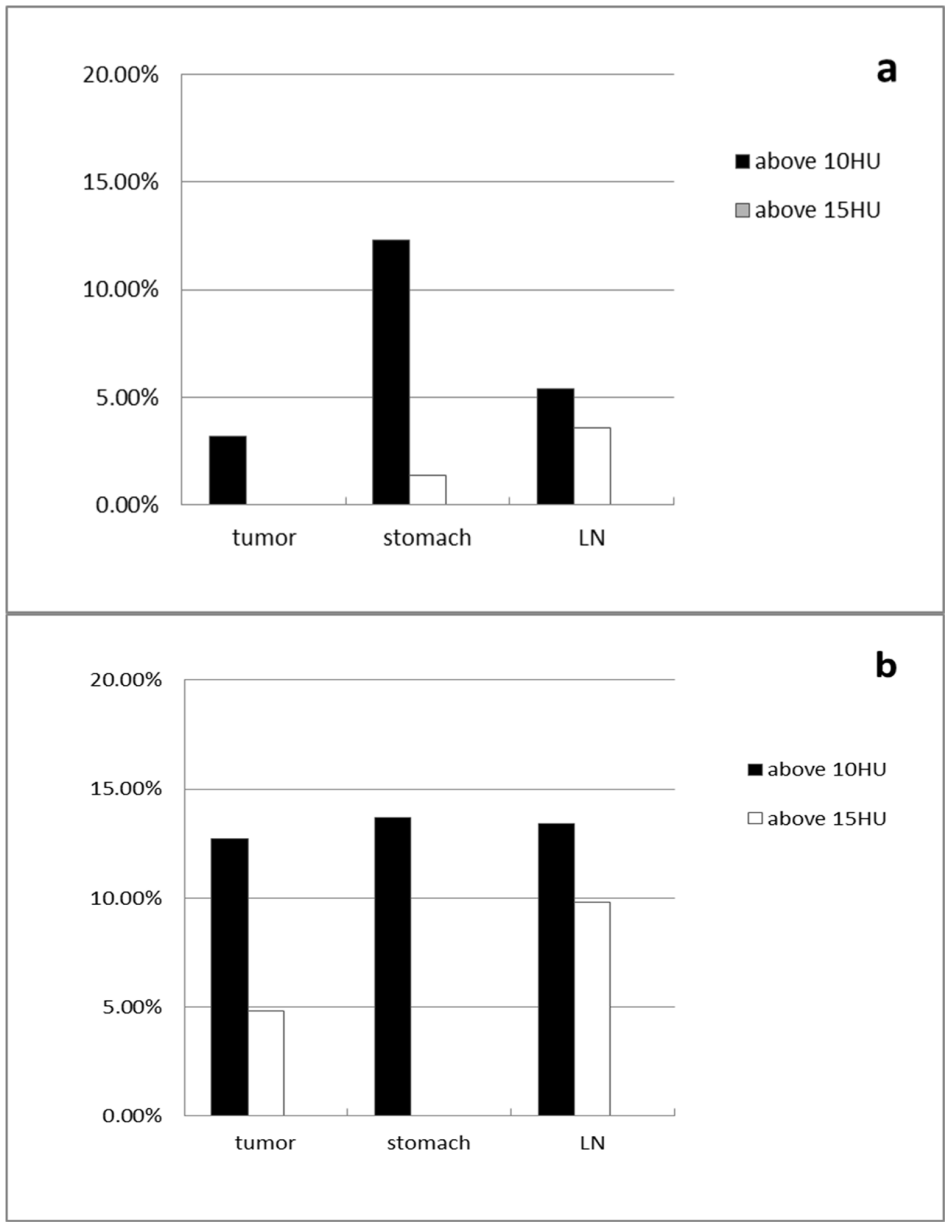
Proportion of measurements stratified per tissue type, with an absolute difference of >10 and >15 Hounsfield units between TNE and VNE images. **a** Proportion of measurements stratified per tissue type, with an absolute difference of >10 and >15 Hounsfield units between TNE and VNE_A_ images. **b** Proportion of measurements stratified per tissue type, with an absolute difference of >10 and >15 Hounsfield units between TNE and VNE_P_ images.

The difference of CT values between TNE and VNE_A_ was under 15 HU in 100%, 98.6%, 96.4%, and was under 10 HU in 96.8%, 87.7%, 94.6% of gastric tumors, stomach wall and enlarged lymph nodes measurements, respectively (Fig. 3a). The CT value difference between TNE and VNE_P_ was under 15 HU in 95.2%, 100%, 90.2%, and was under 10 HU in 87.3%, 86.3%, 86.6%, respectively (Fig. 3b).

**Figure 4:**
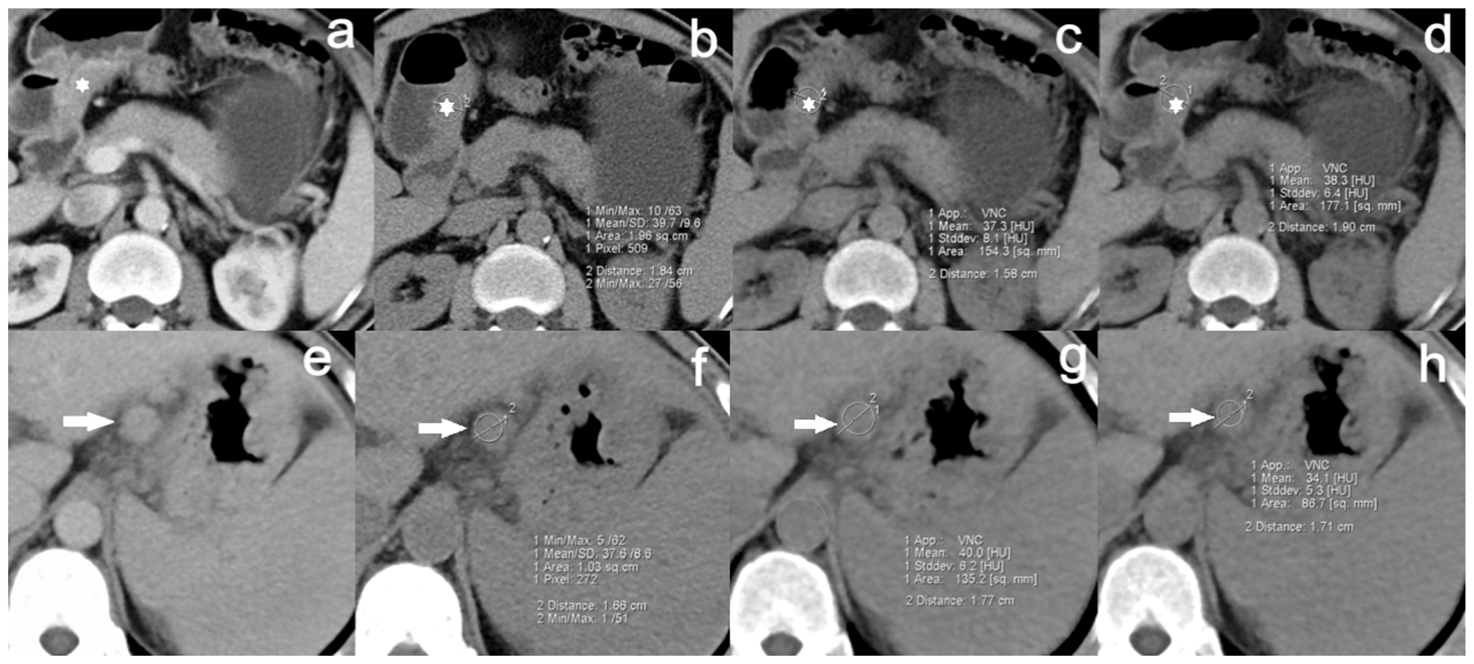
VNE images were acceptable for diagnosis purpose. **a∼d** A 56-year-old man with advanced gastric cancer in the lesser curvature of the antrum. **a** Transverse CT scans show focal wall thickening (asterisk) of the lesser curvature of the gastric antrum with abnormal enhancement. VNE_A_ image (**c**), VNE_P_ image (**d**) and TNE image (**b**) show good correlation of measured CT numbers and thickness of the tumor (TNE: 39.7 HU±9.6, 1.84 cm; VNE_A_: 37.3 HU±8.1, 1.58 cm; VNE_P_: 38.3 HU±6.4, 1.90 cm). **e∼h** A 62-year-old man with advanced gastric cancer in the fundus. **e** Transverse CT scans show an enlarged lymph node (arrow) in the lesser curvature of the gastric body. VNE_A_ image (**g**), VNE_P_ image (**h**) and TNE image (**f**) show good correlation of measured CT numbers and diameter of the node (TNE: 37.6 HU±8.6, 1.66 cm; VNE_A_: 40.0 HU±6.2, 1.77 cm; VNE_P_: 34.1 HU±5.3, 1.71 cm). Both of the patients are noted excellent VNE image quality.

**Figure 5:**
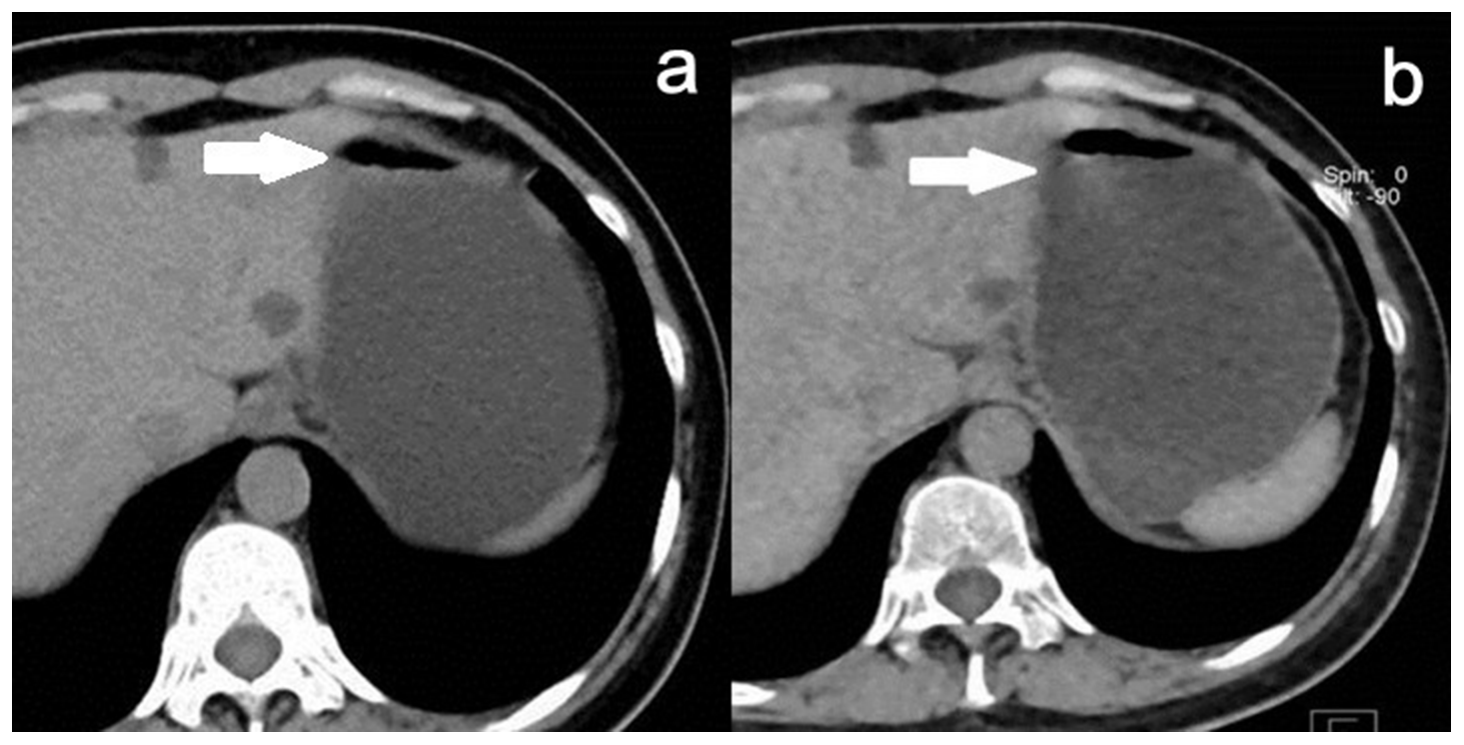
More obvious overshooting artifact at the gas-fluid level (arrow) was detected in VNE image. **a.** TNE image; **b.** VNE image.

**Figure 6:**
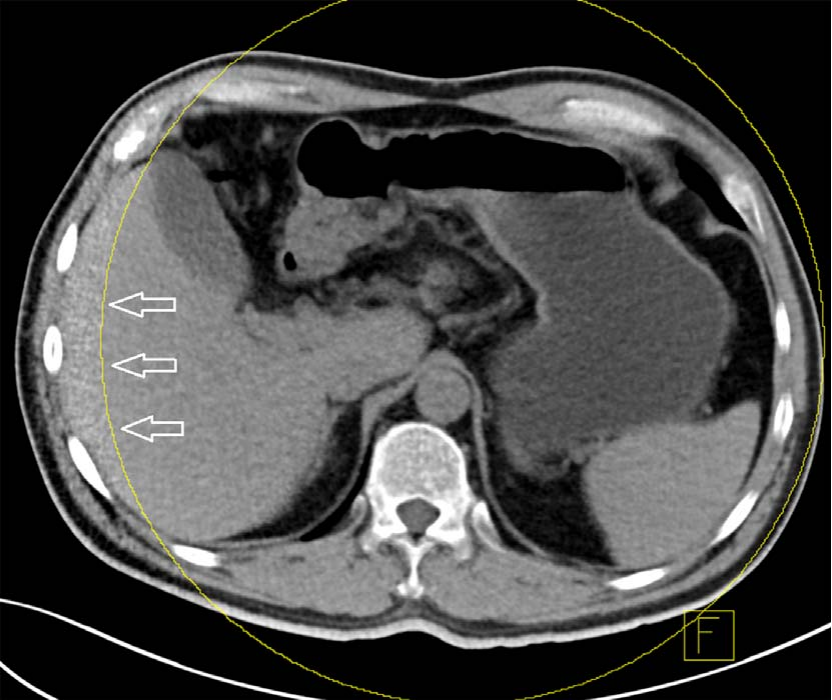
Excluded relevant anatomy. The yellow circle relates to the smaller (33 cm) field of view of the smaller detector. Iodine maps and subsequent iodine subtraction to create the virtual non-enhanced images can only be performed within the circle. In larger patients (>40 cm actual body diameter) or patients with improper position important anatomy may be excluded from the subtraction. In this example the lateral aspect of the right lobe of the liver fails to ‘become unenhanced’ (arrows).

The subjective image quality scores of the three non-enhanced CT image serials (TNE, VNE_A_ and VNE_P_) were 4.93±0.3, 4.15±0.7 and 4.4±0.6, respectively. All VNE images were rated as score 3 or above, indicating that all VNE images were acceptable for diagnosis purpose (Fig. 4). Image quality of both VNE images was lower than that of TNE images (P<0.01), while image quality of VNE_P_ was higher than that of VNE_A_ (P<0.01). Sixty-two (83.8%, 62/74) VNE_A_ images and seventy-one (95.9%, 71/74) VNE_P_ images were regarded as having the potential to replace TNE images by two experienced abdominal radiologists (Table 2). Overshooting artifact detected at the gas-fluid level was more obvious in VNE images than TNE images in 3 cases (Fig. 5). In addition, only one VNE data sets excluded relevant anatomy which was scored 2 (1–25% excluded) (Fig. 6).

**Table 2:** Image quality true non-enhanced and virtual non-enhanced images.

Image quality	TNE	VNE_A_	VNE_P_
1 = not assessable	0	0	0
2 = poor	0	0	0
3 = sufficient	1 (1.4%)	12 (16.2%)	3 (4.1%)
4 = good	3 (4.1%)	39 (52.7%)	36 (48.6%)
5 = excellent	70 (94.6%)	23 (31.1%)	35 (47.3%)
Mean ± SD	4.93±0.3	4.15±0.7	4.4±0.6

TNE true non-enhanced CT, VNE_A_ VNE data acquired at the arterial phase, VNE_P_ VNE data acquired at the portal venous phase.

### Radiation Dose

The calculated effective doses were respectively 2.54±0.79 mSv (range 1.61–6.40 mSv), 3.16±0.91 mSv (range1.79–6.86 mSv), and 6.21±1.63 mSv (3.08–8.99 mSv) for true non-enhanced phase, DE arterial and portal venous phase. For a triple-phase protocol, the total mean dose was 11.92±2.34 mSv (range 7.65–18.48 mSv), while a dual-phase protocol delivers a mean effective dose of 9.37±1.89 mSv (range 4.90–16.53 mSv). The dose reduction achieved by omitting the true non-enhanced acquisition was 21.40±4.44% (range 15.01–38.24%; P<0.01).

## Discussion

The TNE image is necessary in the gastric preoperative CT scan for three reasons. First, the gastric tumor and enlarged lymph nodes require a baseline non-enhanced CT value to calculate the contrast enhancement, which is especially crucial in estimating whether regional lymph nodes represent local metastases or not. Second, enhancing liver metastases may be missed in enhanced images [18]. Third, the calcification and hemorrhage of tumor may also be missed in enhanced images [19].

Dual-energy CT of the abdomen provides two advantages as opposed to single-energy CT: improved image quality of the contrast enhancement itself [10] and the ability to generate VNE images. Compared with TNE images with lower image noise, VNE images are with lower but diagnostic image quality. This is due to particular image filtering and smoothing induced by the post-processing algorithm [7]. Therefore, radiologists can reliably discriminate both types of images. As our results revealed the similar attenuations and morphological feature between VNE and TNE images on detection of gastric cancer, VNE is likely to be regarded as a replacement for TNE scans. When compared with a triple-phase protocol, a dual-phase approach that includes arterial and portal venous phases reduces the effective dose by an average of 21.4%. This is lower than previously published data about the liver and renal images, where a dose reduction of between 30% and 35% has been described [7], [8], [10], as the portal venous phase scan included the whole abdomen and pelvis, from the diaphragmatic domes to the anal verge in our study. DE imaging in patients with gastric tumors is the potential to reduce the effective radiation dose delivered to the patient and also to save investigation time.

Our results suggest that VNE_P_ images had higher subjective score than VNEA images. It should be noted that previous authors have shown VNE_A_ images to be superior by the 1st generation DSCT [8], and VNE_P_ to be better by 2nd generation DSCT [12]. The reason may be due to the fact that the arterially derived series required an additional post-processing step; they were acquired as 1 mm thick slices and needed to be ‘thickened’ to create virtual 2 mm slices [12]. Certainly the use of additional post-processing steps has the potential to degrade the images. There is little to choose between the portal and arterial derived series, however, we recommend the use of the VNE_P_ images over the VNE_A_ images because of the slightly better objective and subjective results and the reduced post-processing time.

As the larger FOV of the smaller detector, which is 33 cm in the second generation DSCT, our study suggested relevant anatomy was considered to be excluded in only one case; in this case that was considered minimal and was <25%. Moreover, a further advantage of gastric DECT is that as a result of the median position of the stomach and the locations of lymphatic metastasis in the abdomen, the smaller FOV hardly affected the gastric preoperative CT scan.

However, with the present technology DECT has several limitations. First, as previous authors have shown, calcium is not among the three materials (soft tissue, iodine, and fat) analyzed in the decomposition process. This limitation can potentially be problematic especially for the detection of mucinous gastric carcinoma [20], [21], but it can be overcome by using another post-processing algorithm that analyses calcium, iodine, and soft tissue [7]. Second, dedicated filtering algorithms can be used to reduce image noise, but again, it may be more difficult to resolve small structures [22]. Third, to further reduce radiation dose from CT scanning, the lesion can be located on TNE images to define the minimum possible scanning range in arterial phase; in addition, the patient can drink more water or change the position after TNE scan, if not good enough distention gastric wall and lesion were found in TNE images. Omitting TNE scan will make these impossible. Finally, overshooting artifact detected at the gas-fluid level was more obvious in VNE images. Though this kind of artifact has not impaired the image quality, but it could represent an important pitfall in some cases. Since this was not among the aims of our study, further analysis is required.

Our study had several limitations. It was performed to prove the quality of VNE images, and not to show the capabilities of DECT in the accuracy of preoperative staging of gastric cancer. Therefore, the diagnostic value of different types of visualization of the DE information, including color coding of iodine distribution, was not assessed in this study. Further research should be recommended to show whether DECT will help depict and accurately preoperative staging in gastric cancer. Second, we did not evaluate the performance of VNE images in detecting liver lesions which is an important route of metastatic gastric cancer. However, in previous reports, VNE images show a quality comparable with that of TNE images, and potential to replace TNE as part of a multiphase liver imaging protocol [8], [10], [12]. Third, since no calcification in stomach tumors was found in our data, the limitation of subtraction of calcium by the Liver VNC algorithm could not be evaluated.

## Conclusions

Our early experience with gastric virtual non-enhanced images using second generation dual-source CT demonstrates promising results. Virtual non-enhanced images generated from either arterial or portal venous phase images provide attenuation values and morphological parameter close to the true non-enhanced images, and demonstrate good image quality. It will be potentially possible to omit TNE scan as part of a multiphase gastric preoperative staging and follow-up study imaging protocol, resulting in significantly reducing the radiation dose.
